# Reversal of HER2 Negativity: An Unexpected Role for Lovastatin in Triple-Negative Breast Cancer Stem Cells

**DOI:** 10.7150/jca.39265

**Published:** 2020-03-31

**Authors:** Huimei Yi, Mi Wu, Qiuting Zhang, Lu Lu, Hui Yao, Sisi Chen, Ying Li, Chanjuan Zheng, Guangchun He, Xiyun Deng

**Affiliations:** 1 Key Laboratory of Translational Cancer Stem Cell Research, Hunan Normal University, Changsha, Hunan 410013, China;; 2 Departments of Pathology and Pathophysiology, Hunan Normal University School of Medicine, Changsha, Hunan 410013, China.

**Keywords:** triple-negative breast cancer, reversal, HER2, cancer stem cell, cancer therapy, lovastatin

## Abstract

Effective treatment modality for triple-negative breast cancer (TNBC) is currently lacking due to the absence of defined receptor targets. Recently, we have demonstrated that lovastatin, a 3-hydroxy-3-methylglutaryl-coenzyme A reductase inhibitor and a lipid-lowering drug, can selectively inhibit TNBC by targeting cancer stem cells *in vivo* and* in vitro*. Interestingly, we found that lovastatin induced the reappearance of human epidermal growth factor receptor 2 (HER2), one of the triple receptors that are missing in TNBC. This prompted us to explore the possibility of regaining sensitivity of TNBC cancer stem cells to receptor tyrosine kinase-targeting drugs. We found that while the combination of lovastatin with a HER2 inhibitor was not sufficient to show synergism, addition of an epidermal growth factor receptor (EGFR/HER1) inhibitor to this combination resulted in significant synergistic inhibitory effect on cell viability. Our findings provide a potential novel strategy of designing a cocktail composed of a lipid-lowering drug and two receptor tyrosine kinase inhibitors for the treatment of TNBC.

## Introduction

Breast cancer is the most common female malignancy and is responsible for about 14% of cancer-related deaths in women 1. Triple-negative breast cancer (TNBC), characterized by the absence of expression of estrogen receptor (ER), progesterone receptor (PR), and human epidermal growth factor receptor 2 (HER2), is the most aggressive and deadly subtype of breast cancer 2. TNBCs constitute 15-20% of all diagnosed breast cancer cases and preferably strike younger patients 3. Tumor characteristics of TNBCs include rare histologies, high grade, and elevated mitotic count, tumor necrosis, pushing margins of invasion, larger tumor size, and axillary node involvement 4. Currently, TNBC patients cannot be treated with endocrine or HER2-targeting therapies used for non-TNBC patients. Although initially responsive to chemotherapy, TNBC patients relapse more frequently than those with other subtypes of breast tumors 4, 5. Therefore, understanding the biology of the missing receptors and exploring the possibilities of their reactivation become extremely crucial both on the bench and in the clinic, with the hope of regaining sensitivity for TNBC cells to receptor-targeting drugs.

Lovastatin (LV), a natural 3-hydroxy-3-methylglutaryl-coenzyme A (HMG-CoA) reductase inhibitor, is one of the most commonly used lipid-lowing drugs 6. The anti-cancer properties of statins have attracted increasing interest over the last decades 7. Our recent studies have demonstrated that LV can inhibit TNBC by targeting cancer stem cells (CSCs) both *in vivo* and *in vitro* 8, 9. However, it is not known whether LV is able to reverse the triple-negative phenotype in TNBC cells. In this study, we unexpectedly found that LV could induce the reappearance of HER2, one of the missing receptors in TNBC cells. This prompted us to examine whether this reversal of receptor-negative phenotype could sensitize these cells to the drugs that target the receptor tyrosine kinases. Our pilot study provides evidence that the receptor tyrosine kinase inhibitors that specifically target HER2 and epidermal growth factor receptor (EGFR/HER1), respectively, could act synergistically to suppress the cell viability of LV-challenged TNBC CSCs. These findings provide a potential strategy of combination therapy for TNBC that deserves further investigations in more sophisticated model systems.

## Lovastatin induces the reappearance of HER2 in TNBC CSCs

**Figure 1:**
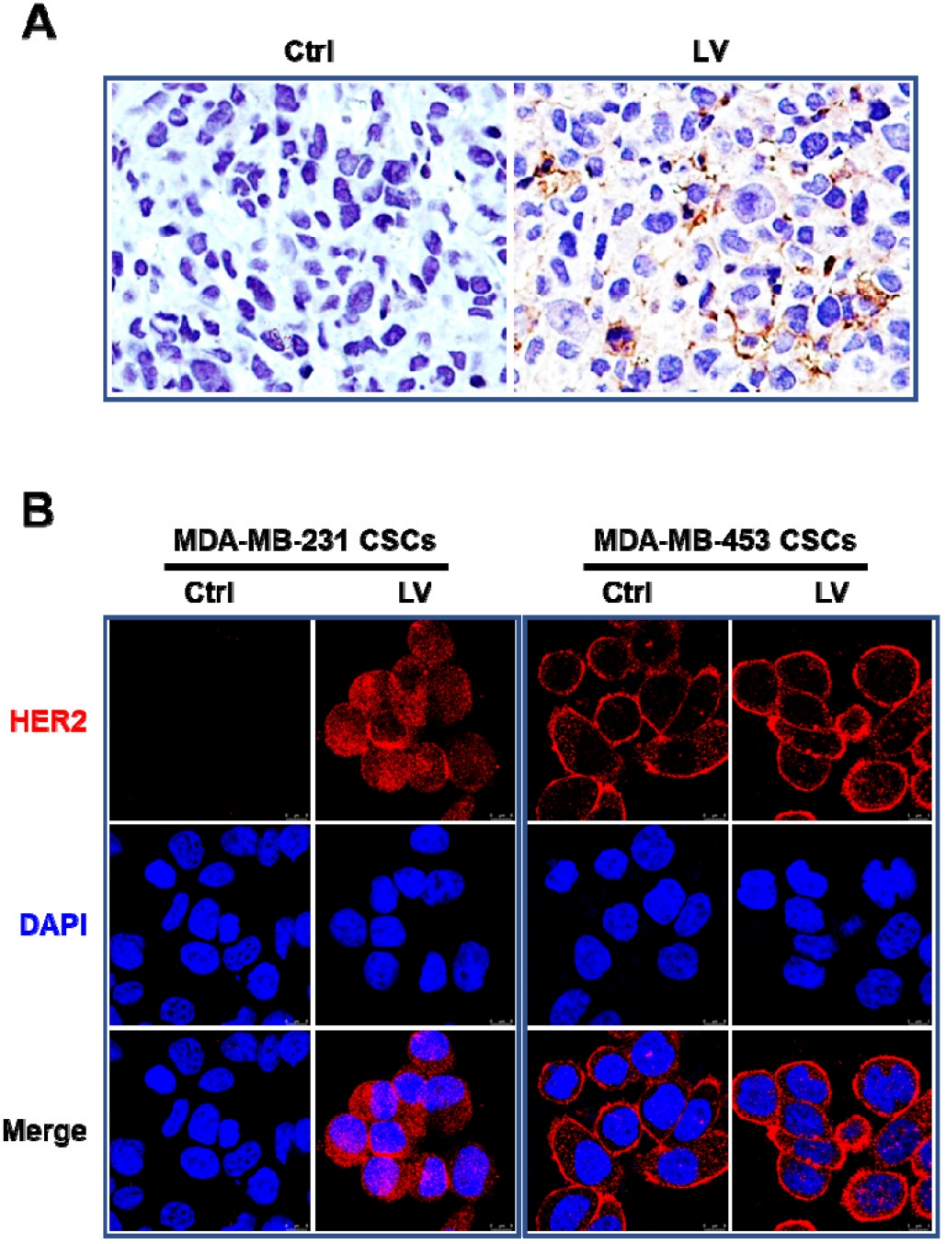
** Effect of lovastatin on HER2 in TNBC CSCs *in vivo* and *in vitro*. A.** Representative images showing immunohistochemical staining of HER2 in the nude mouse model of orthotopic tumors derived from TNBC MDA-MB-231 CSCs. Original magnification: 40 ×.** B.** Representative laser confocal microscopic images showing immunofluorescence staining of HER2 in TNBC CSCs and non-TNBC CSCs. MDA-MB-231 (TNBC) and MDA-MB-453 (HER2-positive) CSCs were treated with LV (1.0 µM) or vehicle control for 48 h. DAPI was used to stain the nucleus (blue). Scale bar = 8 µm. Original magnification: 63 ×. LV: lovastatin; Ctrl: control; CSCs: cancer stem cells.

In order to determine whether LV could reverse the triple-negative phenotype of TNBC, we first performed immunohistochemistry to evaluate the status of HER2 and ER in the nude mouse model of orthotopic tumor growth derived from mammary fat pad injection of TNBC MDA-MB-231 CSCs. We found that LV induced the reappearance of HER2 in the tumor tissues **(Figure 1A)**. LV also induced the reappearance of ER in the same mouse model (data not shown). In cultured MDA-MB-231 CSCs, LV-induced reappearance of HER2 was demonstrated by immunofluorescence-laser scanning confocal microscopy **(Figure 1B)**. However, unlike the solely membranous distribution in non-TNBC MDA-MB-453 CSCs, LV-induced HER2 in TNBC CSCs showed a distinct pattern of both membranous and cytoplasmic distribution. This suggests that LV-induced HER2 might be different from the prototype HER2 in HER2-positive cells.

## Targeting of both HER2 and EGFR/HER1 signaling synergistically inhibits the viability of lovastatin-treated TNBC CSCs

**Figure 2:**
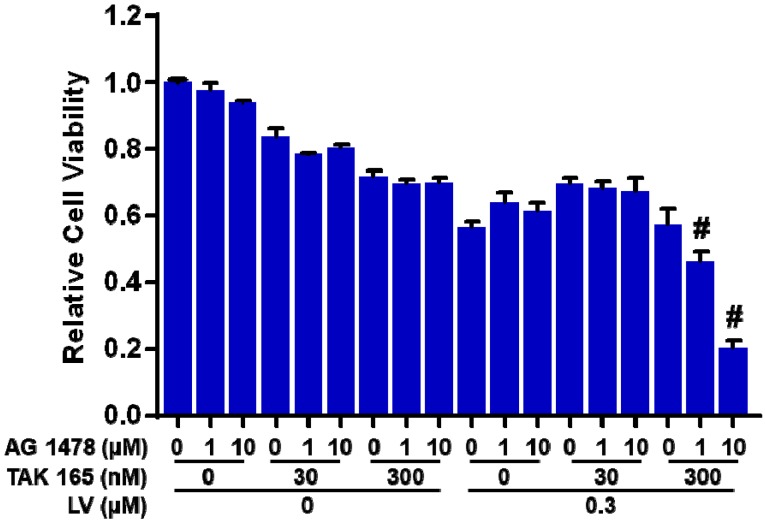
** Effect of lovastatin, HER2 inhibitor and HER1 inhibitor on the cell viability of TNBC CSCs.** MDA-MB-231 CSCs were treated with different concentrations of LV, TAK 165 (HER2 inhibitor) and/or AG 1478 (HER1 inhibitor) for 72 h. The cell viability was detected by AlamarBlue assay. Combination index (CI) was calculated using the CompuSyn software. CI < 1.0 (#) indicated a synergistic effect.

To explore the possibility whether LV-induced HER2 could sensitize TNBC CSCs to HER2-targeting drugs, we combined LV with a HER2 inhibitor (TAK 165) to treat TNBC CSCs. Unfortunately, this combination did not synergistically inhibit the cell viability of TNBC CSCs. Considering the fact that EGFR/HER1 signaling pathway is activated in ER-negative breast cancer 10, 11, we next added an EGFR/HER1 inhibitor (AG 1478) to the combination of LV and HER2 inhibitor to treat TNBC CSCs. As expected, this cocktail could act synergistically to inhibit the cell viability of TNBC CSCs **(Figure 2)**. These results highlight the importance of inhibition of both HER2 and EGFR/HER1 signaling in eradicating TNBC CSCs that survive LV treatment.

## Perspectives

Recently, we have demonstrated that LV, by itself, could induce death of TNBC CSCs through induction of stress response pathways 12 and inhibition of stemness properties (data not shown). It is likely that at the concentration used (1 µM), LV causes the death of some cells, while sparing the rest of the cells with HER2 signaling pathway reactivated. Reactivation of this life-sustaining signaling pathway confers the cells the capability to survive the harsh environment such as stressor challenge. In relation to the triple negativity of TNBC, the activation of HER2 signaling reverses the receptor-negative phenotype. It is hoped that the reappearance of the missing receptor should render the cells sensitive to receptor-targeting therapies, which will have a great clinical impact on the treatment of TNBC.

**Figure 3:**
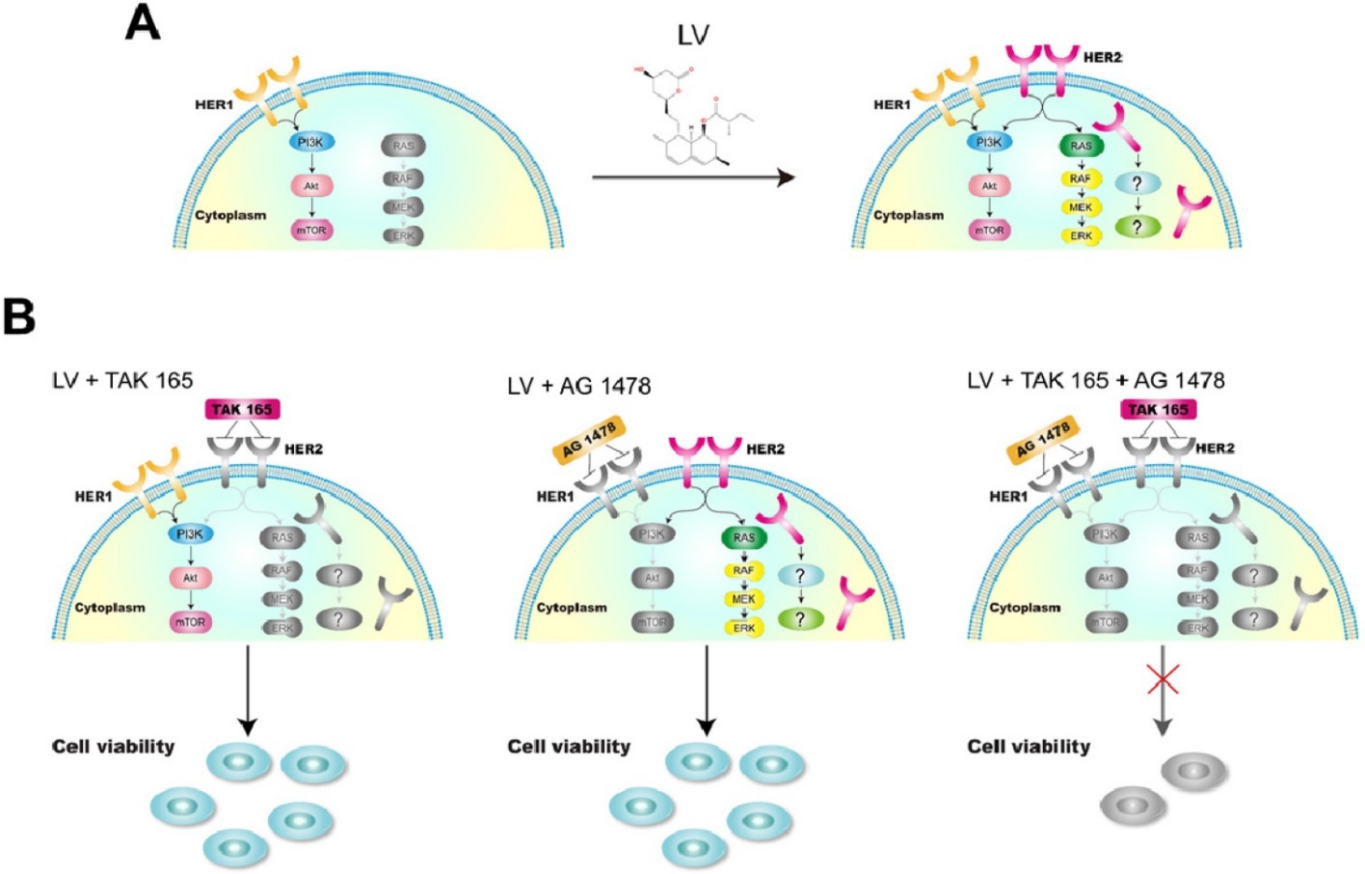
** A model showing the reversal of HER2 negativity by lovastatin and regaining the sensitivity to tyrosine kinase-targeting drugs in TNBC CSCs. A.** LV induces the reappearance of HER2 in TNBC CSCs, which shows a distinct pattern of membranous and cytoplasmic distribution. **B.** LV in combination with TAK 165 (HER2 inhibitor) or AG 1478 (HER1 inhibitor) or both shows differential effects on TNBC CSCs. While the combination of LV with TAK 165 or AG 1478 separately does not lead to suppression of cell viability, the combination of LV with both TAK 165 and AG 1478 together shows synergistically inhibitory effect on TNBC CSCs. The drawings in color and grey scale represent active and inactive signaling pathways, respectively.

In our hands, although the combination of LV with a HER2 inhibitor had no synergistically inhibitory effect on TNBC CSCs, the cocktail composed of LV and two receptor tyrosine kinase inhibitors, i.e., a HER2 inhibitor (TAK 165) and an EGFR/HER1 inhibitor (AG 1478), could synergistically inhibit the cell viability. We chose to use these inhibitors as a proof of concept because they are more specific for the individual signaling pathway than the commonly used dual-specificity receptor tyrosine kinase inhibitors such as lapatinib. In the case of TNBC CSCs, simultaneous inhibition of the complex intracellular signaling pathways, e.g., multiple receptor signaling and stemness-sustaining signaling, might be necessary in eliminating CSCs **(Figure 3)**. Since HER2 signaling is related to cell proliferation and/or survival, the reactivation of this pathway is of utmost importance in designing therapeutic strategy targeting cell proliferation and survival. In the future, we need to investigate the impact of the reversal of receptor negativity on cell behavior in more sophisticated model systems, including models of cancer cell implantation or patient-derived xenografts. We are far away from a clear view of the whole picture of the reversal of receptor negativity and its clinical implications. Nevertheless, this possibility provides a valuable opportunity for us to explore to fight against the difficult-to-treat TNBC.

## Abbreviations

TNBC: Triple-negative breast cancer
HMG-CoA: 3-hydroxy-3-methylglutaryl-coenzyme A
ER: Estrogen receptor
PR: Progesterone receptor
HER2: Human epidermal growth factor receptor 2
HER1: Human epidermal growth factor receptor 1
EGFR: Epidermal growth factor receptor
LV: Lovastatin
CSC: Cancer stem cell
Ctrl: Control
CI: Combination index
